# Construction of a Nomogram for Portal Vein Thrombosis After Endoscopic Therapy of Severe Gastroesophageal Varices in Cirrhotic Patients

**DOI:** 10.5152/tjg.2025.24398

**Published:** 2025-07-16

**Authors:** Jiamei Zhou, Lingyun Niu, Xinyu Chen, Tao Han, Huiling Xiang, Hao Cui, Baiguo Xu, Jing Liang, Jia Lian, Ping Zhu, Junqing Yan

**Affiliations:** 1 Department of Gastroenterology, Second Central Hospital of Baoding, Hebei, China; 2 Tianjin Union Medical Center, Tianjin Medical University, Tianjin, China; 3 Department of Gastroenterology, Shanxi Bethune Hospital, Shanxi Academy of Medical Sciences, Third Hospital of Shanxi Medical University, Tongji Shanxi Hospital, Taiyuan, China; 4 Tianjin Key Laboratory of Extracorporeal Life Support for Critical Diseases, Institute of Hepatobiliary Disease, The Third Central Clinical College of Tianjin Medical University, Tianjin University Central Hospital (Tianjin Third Central Hospital), Tianjin, China; 5 Department of Hepatology and Gastroenterology, Tianjin Union Medical Center, Tianjin Medical University, Tianjin, China; 6 Department of Gastroenterology and Hepatology, Tianjin Key Laboratory of Extracorporeal Life Support for Critical Diseases, Institute of Hepatobiliary Disease, Tianjin University Central Hospital (Tianjin Third Central Hospital), Tianjin, China

**Keywords:** Cirrhosis, endoscopic variceal treatment, inflammation markers, nomogram, portal vein thrombosis, risk factors

## Abstract

**Background/Aims:**

Limited research exists on predictors of portal vein thrombosis (PVT) during endoscopic therapy. This study aims to develop a nomogram, utilizing inflammatory markers, to predict the development of PVT within 2 years following endoscopic treatment in cirrhotic patients with severe gastroesophageal varices.

**Materials and Methods:**

A retrospective analysis was conducted on clinical data from 127 patients who underwent endoscopic therapy for severe gastroesophageal varices or bleeding due to cirrhosis at Tianjin Third Central Hospital between January and December 2015, all of whom were free of PVT at baseline. Univariate and multivariate logistic regression models were applied to identify significant predictors. A nomogram for PVT occurrence was developed.

**Results:**

Of the 127 patients, 20 developed PVT (PVT group), representing an incidence rate of 15.75% within 2 years. The remaining 107 patients did not develop PVT (non-PVT group). Multivariate logistic regression revealed that the model for end-stage liver disease score, monocyte-to-lymphocyte ratio (MLR), spleen diameter, and gastric varices sandwich therapy were independent predictors of PVT development. A nomogram was constructed, with the area under the receiver operating characteristic (ROC) curve (AUC) of 0.807 (95% CI: 0.703-0.911, *P* = .000) and a C-index of 0.807. The Hosmer-Lemeshow *χ*^2^ test score was 3.681 (*P* = .885). Bootstrap self-sampling confirmed the model’s excellent calibration, while the decision curve analysis (DCA) indicated a higher net benefit at higher risk threshold probabilities.

**Conclusion:**

A nomogram predicting PVT development in cirrhotic patients with severe gastroesophageal varices within 2 years of the first endoscopic intervention was successfully established using inflammatory markers. This model offers an effective tool for predicting PVT risk in this patient population.

**Main Points**

- Increasing evidence suggests a strong association between inflammatory markers and the onset of portal vein thrombosis (PVT). This study identified the monocyte-to-lymphocyte ratio as a significant predictor for PVT development in cirrhotic patients with severe gastroesophageal varices undergoing endoscopic therapy.
- Splenomegaly is a key indicator of portal hypertension in cirrhosis, with spleen diameter emerging as a crucial risk factor for PVT.
- Among various endoscopic treatments, gastric varices sandwich therapy alone was found to be an independent factor influencing the occurrence of PVT.

## Introduction

Portal vein thrombosis (PVT) refers to the formation of thrombosis in the main portal vein and/or its left and right branches, with or without the involvement of the mesenteric and splenic veins.^1^ Initially considered a rare complication in cirrhotic patients due to its subtle clinical presentation, the diagnostic rate has increased as awareness among clinicians has grown. Endoscopic variceal treatment is a key intervention for preventing and managing esophagogastric variceal bleeding in cirrhosis. However, endoscopic treatment may elevate the risk of PVT in these patients.^2^^-^^4^ Portal vein thrombosis exacerbates the risk of esophagogastric variceal bleeding, prolongs variceal elimination, and increases the incidence of complications such as hydrothorax and ascites.^5^^,^^6^ The insidious onset of PVT often leaves patients without prominent clinical symptoms. Identifying high-risk individuals early in cirrhotic patients undergoing endoscopic therapy, facilitating early detection, and timely intervention have become critical in improving the prognosis of these patients. Early management can shorten treatment duration and reduce the economic burden on patients.

Inflammatory markers, including the monocyte-to-lymphocyte ratio (MLR), systemic immune-inflammation index (SII), neutrophil-to-lymphocyte ratio (NLR), and platelet-to-lymphocyte ratio (PLR), are predictive of venous thrombosis in conditions such as deep vein thrombosis (DVT),^7^^-10^ cerebral venous thrombosis,^11^ and cancer-related thrombosis.^12^^,^^13^ One study revealed that a nomogram based on MLR could help clinicians assess the likelihood of DVT early.^14^ Other research has indicated that MLR, PLR, and NLR have predictive value for PVT in cirrhotic patients undergoing splenectomy.^15^^,^^16^ Additionally, a nomogram model based on MLR has been shown to effectively predict the risk of PVT in cirrhotic patients after splenectomy combined with esophagogastric devascularization.^17^ This study aims to explore the factors influencing PVT occurrence within 2 years after the initial endoscopic treatment for severe gastroesophageal varices in cirrhotic patients, assess the predictive value of inflammatory markers, and develop a nomogram prediction model for PVT.

## Materials and Methods

### Patients

Patients with severe gastroesophageal varices or rupture bleeding associated with cirrhosis who were admitted to the Department of Gastroenterology from January to December 2015 for their first endoscopic treatment were selected for this study. The observation period began at the time of the initial gastroscopic treatment, and imaging exams were conducted every 3 months. Portal vein thrombosis occurrence was monitored during the 2-year follow-up. All patients or their family members provided written informed consent prior to undergoing endoscopic treatment.

Inclusion criteria: (1) cirrhosis confirmed through medical history, physical examination, laboratory tests, imaging, and pathological evaluation;^18^ (2) severe gastroesophageal varices confirmed by gastroscopy;^19^ (3) first endoscopic therapy performed to prevent initial bleeding or control acute bleeding; and (4) imaging (abdominal color ultrasound or contrast-enhanced computed tomography) performed during the first hospitalization to confirm the absence of PVT.

Exclusion criteria: (1) patients without imaging confirming the absence of PVT upon admission; (2) presence of malignant tumors; (3) presence of thrombotic or hematopoietic disorders; (4) severe dysfunction of other organs; (5) prior use of anticoagulants; (6) history of splenectomy, interventional embolization, hemostasis, transjugular intrahepatic portosystemic shunt, radiofrequency ablation, or liver transplantation before or during follow-up; (7) concomitant Budd-Chiari syndrome; and (8) patients who did not complete follow-up due to liver cancer, other malignancies, death, or loss to follow-up. This study was approved by the Ethics Committee of University of The Third Central Clinical College of Tianjin Medical University (approval number: IRB2019-040-01, date: April 1, 2019).

### Observation Indicators

Clinical data collected prior to the first endoscopic treatment included patient demographics (age, sex), smoking and drinking history, cirrhosis etiology, and history of ascites, type 2 diabetes, hypertension, spontaneous bacterial peritonitis (SBP), and hepatic encephalopathy (HE). Auxiliary test results included albumin (ALB), white blood cells (WBC), globulin (GLB), neutrophils (NE), monocytes (MO), total bilirubin (TBIL), lymphocytes (LYM), platelets (PLT), red blood cell distribution width, mean platelet volume, portal vein diameter, blood urea nitrogen, spleen thickness and diameter, international normalized ratio (INR), creatinine (Cr), and prothrombin time (PT). Data on the purpose of the first endoscopic treatment and subsequent treatments before PVT or during the 2-year follow-up period were also recorded.

### Treatment Method

Patients presenting with esophagogastric variceal bleeding upon admission were initially treated with proton pump inhibitors, somatostatin, and antibiotics. Among the alcoholic cirrhosis patients, all were male, with a significant history of alcohol consumption, characterized by more than 5 years of drinking and an average daily intake exceeding 40 g of ethanol. This drinking pattern and duration met the diagnostic criteria for alcoholic cirrhosis. These patients did not exhibit severe alcoholic heart disease and were managed with alcohol abstinence, liver protection, and nutritional support therapy.

Treatment for esophageal varices was based on 3 approaches: ligation therapy, sandwich therapy, or a combination of ligation and sandwich therapy. In cases of concomitant gastric varices, sandwich therapy was utilized. The sandwich treatment involved the sequential injection of lauromacrogol, cyanoacrylate, and lauromacrogol. After the initial endoscopic treatment, a follow-up gastroscopy was performed 2-4 weeks later to assess the therapeutic effect. Subsequent treatments were administered at intervals of 1-4 months, with multiple cycles of sequential therapy until varices were eliminated or further bleeding risk was mitigated.

### Clinical Scoring and Calculation of Inflammatory Markers

The Child-Turcotte-Pugh (CTP) score was calculated based on the following parameters: HE, ascites, ALB, PT, and TBIL.

The MELD score was calculated using the following formula: MELD score = 3.78 × Ln[TBIL (mg/dL)] + 11.2 × Ln[INR] + 9.57 × Ln[Cr (mg/dL)] + 6.43 × (Etiology of liver cirrhosis: 0 for cholestatic or alcoholic, 1 for others).

Platelet-to-lymphocyte ratio was determined by dividing the absolute platelet count by the lymphocyte count (10^9^/L). Neutrophil-to-lymphocyte ratio represented the absolute neutrophil count divided by the lymphocyte count (10^9^/L). Monocyte-to-lymphocyte ratio was calculated by dividing the absolute monocyte count by the lymphocyte count (10^9^/L), while MPR was the mean platelet volume divided by the platelet count (10^9^/L). RPR denoted the percentage of red cell distribution width divided by platelet count (10^9^/L), and the SII was calculated as the platelet count × neutrophil count / lymphocyte count (10^9^/L). The prognostic nutritional index (PNI) was calculated as albumin (g/L) + 5 × total lymphocyte count (10^9^/L).

### Diagnosis of Portal Vein Thrombosis

The diagnostic methods for PVT include abdominal color ultrasound and contrast-enhanced computed tomography, which detect venous thrombosis in any segment of the main portal vein, its left and right branches, the superior mesenteric vein, or the splenic vein. Diagnosis follows expert consensus on the management of PVT in cirrhosis.^1^

### Statistical Analysis

Statistical analysis was performed using SPSS 26.0 (IBM SPSS Corp.; Armonk, NY, USA) and R version 4.1.3 (Company; City, Country). Missing data were addressed through mean imputation. Normally distributed quantitative data are presented as mean ± SD, with significance assessed via *t*-test. Non-normally distributed data are presented as median and interquartile range, with significance determined using the Mann–Whitney *U* test. Categorical data are expressed as n(%), with significance assessed using *χ*^2^ or Fisher’s exact test. The optimal cutoff value was determined using the Youden index. Variables with *P* < .150 in univariate analysis were included in multivariate logistic regression,^20^ and a nomogram prediction model was developed. Model performance was assessed *via* the area under the receiver operating characteristic (ROC) curve (AUC) and the concordance index (C-index). Calib rati on was evaluated using the Hosmer–Lemeshow test and calibration curve. Internal validation was performed using bootstrap sampling (1,000 iterations). The clinical utility of the model was assessed using Clinical Decision Curve Analysis (DCA). Statistical significance was set at *P* < .05.

## Results

### Clinical Characteristics of the Study Population at Baseline

A total of 127 patients were included based on the inclusion criteria. Among them, 20 patients developed PVT within 2 years, categorized into the PVT group. The remaining 107 patients did not develop PVT and were assigned to the non-PVT group. The incidence of PVT was 15.75% (20 cases/127 cases × 100%). The PVT group consisted of 15 males and 5 females, with a male-to-female ratio of 3:1, and ages ranging from 36 to 76 years (mean age: 55.25 ± 10.58). The non-PVT group included 65 males and 42 females, with a male-to-female ratio of 1.55:1, and ages ranging from 31 to 81 years (mean age: 55.59 ± 11.00).

No significant differences were observed between the groups in terms of sex, age, smoking, drinking history, CTP classification, type 2 diabetes history, hypertension, SBP, HE, ascites, or the purpose of the first endoscopic treatment (*P* > .05). The MELD score in the PVT group was significantly lower than that in the non-PVT group (*P* = .030).

No significant differences were found between the groups in the distribution of hepatitis C cirrhosis, autoimmune cirrhosis, or unknown etiology (*P* > .05). The proportion of hepatitis B cirrhosis patients was higher in the non-PVT group than in the PVT group, but the difference was not statistically significant (*P* = .145). Alcoholic cirrhosis was more prevalent in the PVT group, with a statistically significant difference (*P* = .029, Table 1).

### Portal Vein Thrombosis Occurrence Time, Location Characteristics, Staging, and Severity

The time to PVT occurrence following the first endoscopic varicose vein treatment ranged from 2.5 to 23.5 months (mean: 12.45 ± 6.49). Of the 20 PVT patients, 4 (20%) developed PVT within 6 months, 5 (25%) within 6-12 months, 7 (35%) within 12-18 months (the most common period), and 4 (20%) within 18-24 months.

The main portal vein and its branches were most frequently affected, with 13 cases (65%) involving these areas. Thrombosis also affects the splenic and superior mesenteric veins, either alone or in combination with the portal vein. Of the 20 PVT cases, 13 (65%) involved the main portal vein and its branches, 4 (20%) affected the splenic vein, 1 (5%) affected the superior mesenteric vein, and 2 cases (10%) involved a combination of the portal vein with either the superior mesenteric or splenic vein.

All 20 PVT patients were asymptomatic. Regarding thrombosis severity, partial PVT was the most common, occurring in 14 patients (70%), while mural thrombosis was observed in 6 patients (30%).

### Comparison of Endoscopic Treatment Between Two Groups During Follow-up

No significant differences were observed between the groups in the number of patients treated with esophageal variceal ligation (EVL) alone, esophageal variceal ligation + esophageal variceal sandwich (EVS), or esophageal variceal ligation + gastric variceal sandwich (GVS) (*P* > .05). The PVT group had a higher number of patients treated with GVS alone compared to the non-PVT group, but this difference was not statistically significant (*P* = .117, Table 2).

### Univariate and Multivariate Analyses

No significant differences were found in baseline values for WBC, MO, LYM, NE, PLT, PLR, NLR, PNI, SII, MPR, RPR, HGB, ALB, GLB, TBIL, Cr, PT, INR, portal vein width, or spleen thickness between the 2 groups (*P* > .05). However, the MLR value in the PVT group was significantly higher than in the non-PVT group (*P* = .035). Additionally, the spleen diameter in the PVT group was greater than that in the non-PVT group, with a statistically significant difference (*P* = .008) (Table 3).

Six independent variables from the univariate analysis—hepatitis B cirrhosis, alcoholic cirrhosis, MELD score, MLR, gastric varices sandwich therapy alone, and spleen diameter—were included in the multivariate logistic regression analysis. The analysis identified MELD score, MLR, spleen diameter, and gastric varices sandwich therapy alone as independent factors for predicting PVT occurrence (Table 4).

The prediction model equation is as follows: −8.823-0.205×MELD score+1.000×MLR+ 0.452×spleen diameter (cm) +2.936×gastric varices sandwich therapy alone.

### Cut-off Value of Spleen diameter, Monocyte-to-Lymphocyte Ratio, and Model for End-Stage Liver Disease Score

**Figure 1.**
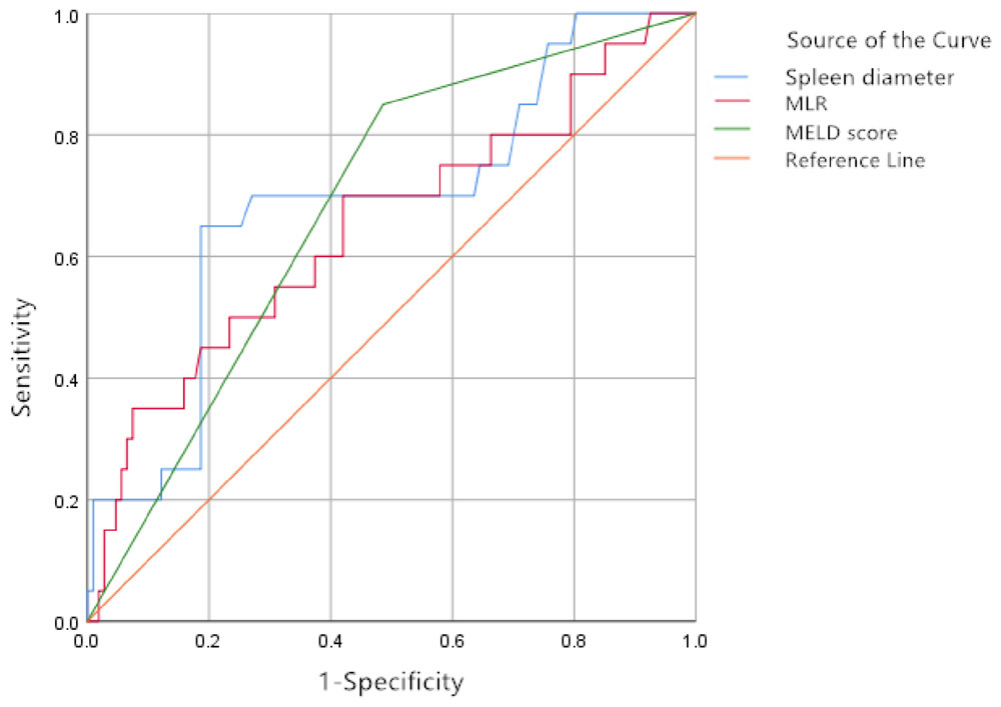
Receiver operating characteristic curves of spleen diameter, monocyte-to-lymphocyte ratio, and model for end-stage liver disease score.

**Figure 2.**
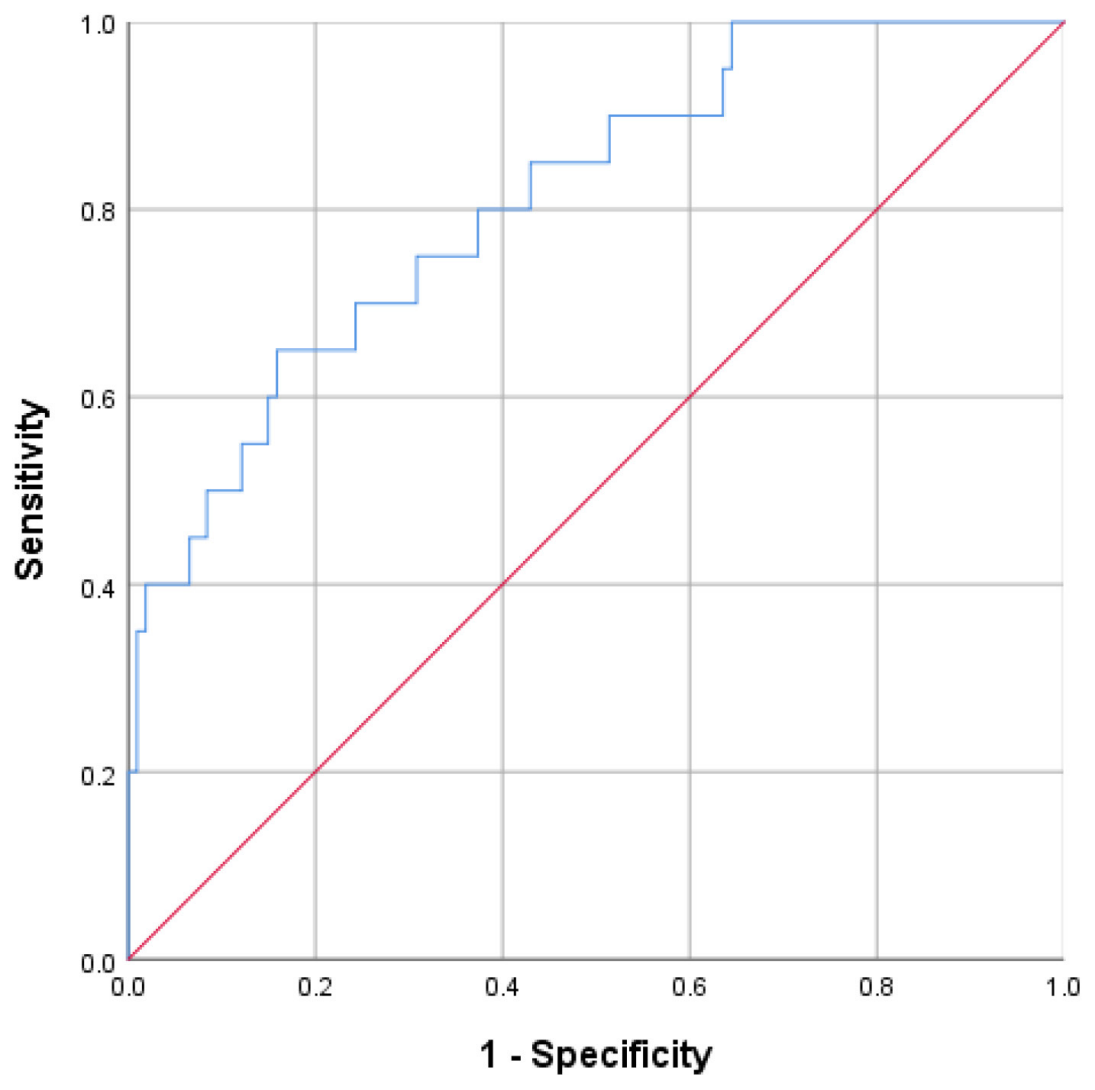
Rreceiver operating characteristic curve of the 3 indexes combined to predict portal vein thrombosis occurrence.

The cut-off values were determined as follows: spleen diameter ≥ 17.135 cm (AUC: 0.688, *P* = .008), MLR ≥ 0.475 (AUC: 0.649, *P* = .035), and MELD score ≥ 9.500 (AUC: 0.682, *P* = .010). The combination of these 3 indexes (spleen diameter + MLR + MELD score) achieved the highest AUC value of 0.782. Furthermore, spleen diameter and MLR demonstrated the highest specificity (92.5%) (Table 5, Figures 1 and 2).

### Nomogram Development

**Figure 3.**
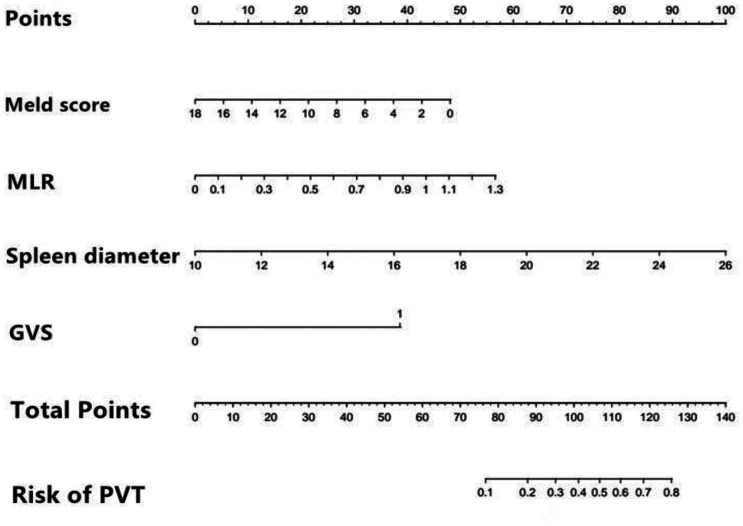
Nomogram of the risk of portal vein thrombosis within 2 years after endoscopic treatment for gastroesophageal varices in cirrhotic patients.

Upon integrating these 4 independent risk factors into the prediction model, an individualized nomogram for predicting the risk of PVT after endoscopic treatment of cirrhosis was developed. Each variable’s score is represented by a vertical line along its respective axis, with the total score derived from the “total value axis.” The corresponding risk of PVT occurrence can be determined by drawing a vertical line down from the total score (Figure 3).

### Nomogram Validation

**Figure 4.**
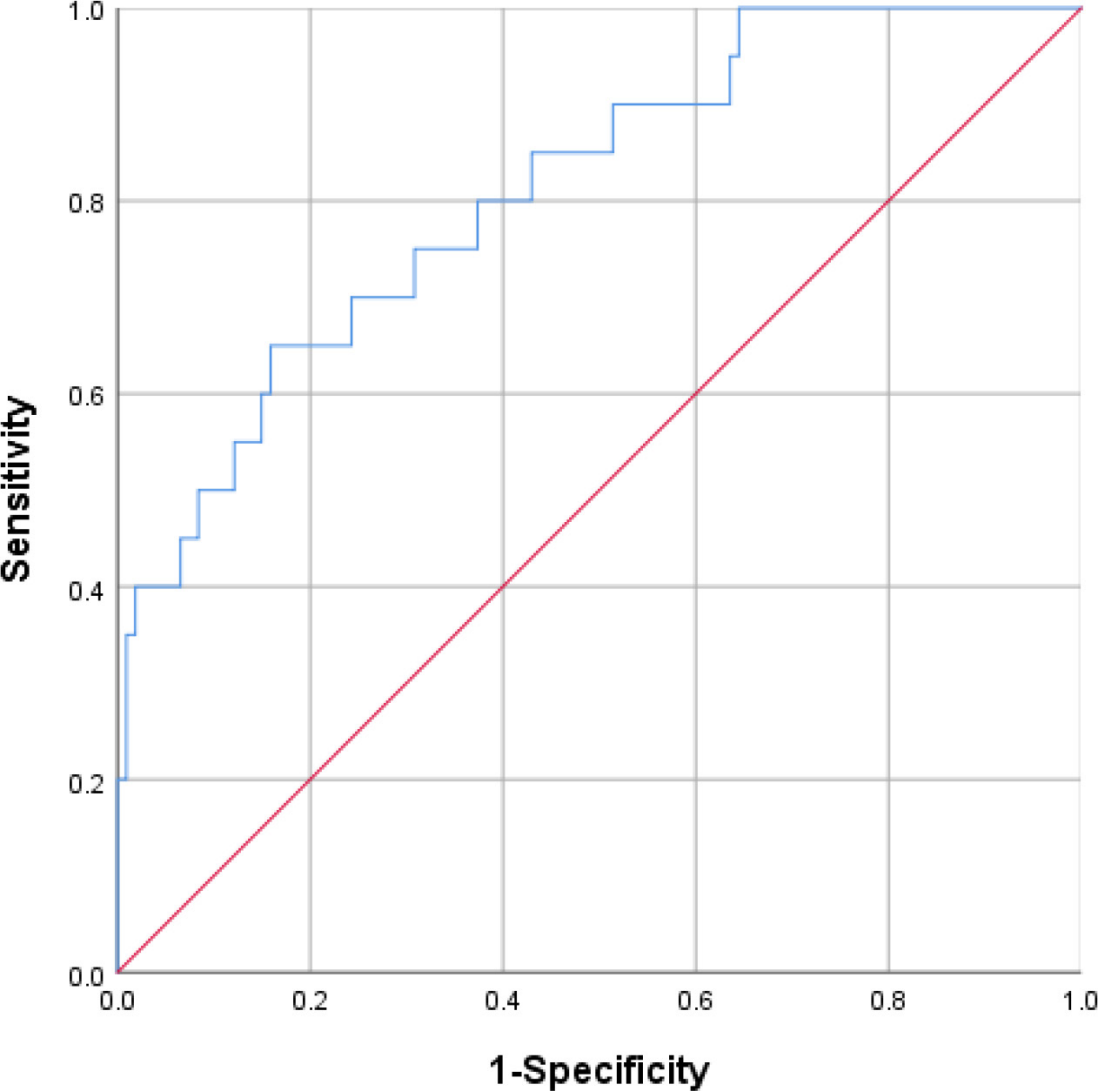
Receiver operating characteristic curve of the model.

**Figure 5.**
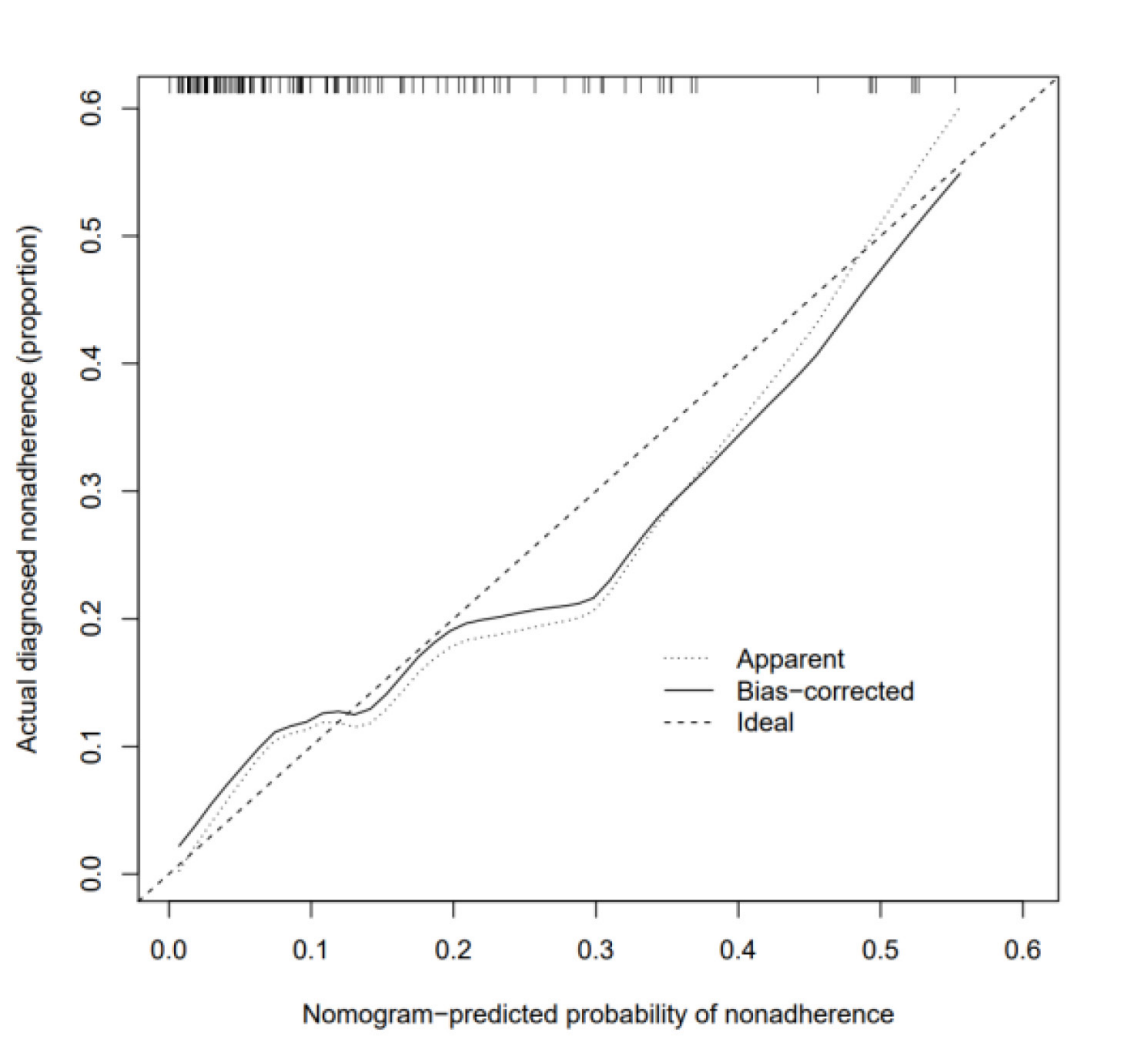
Calibrati on plots of the nomogram.

The AUC value of the model reached 0.807 (95% CI: 0.703-0.911, *P* = .000) (Figure 4), with a concordance index of 0.807. The Hosmer–Lemeshow test revealed a *χ*^2^ value of 3.681 (*P* = .885). Internal validation through the bootstrap method showed that the prediction model’s solid line was closely aligned with the virtual slash line, indicating good calibration (Figure 5).

**Figure 6.**
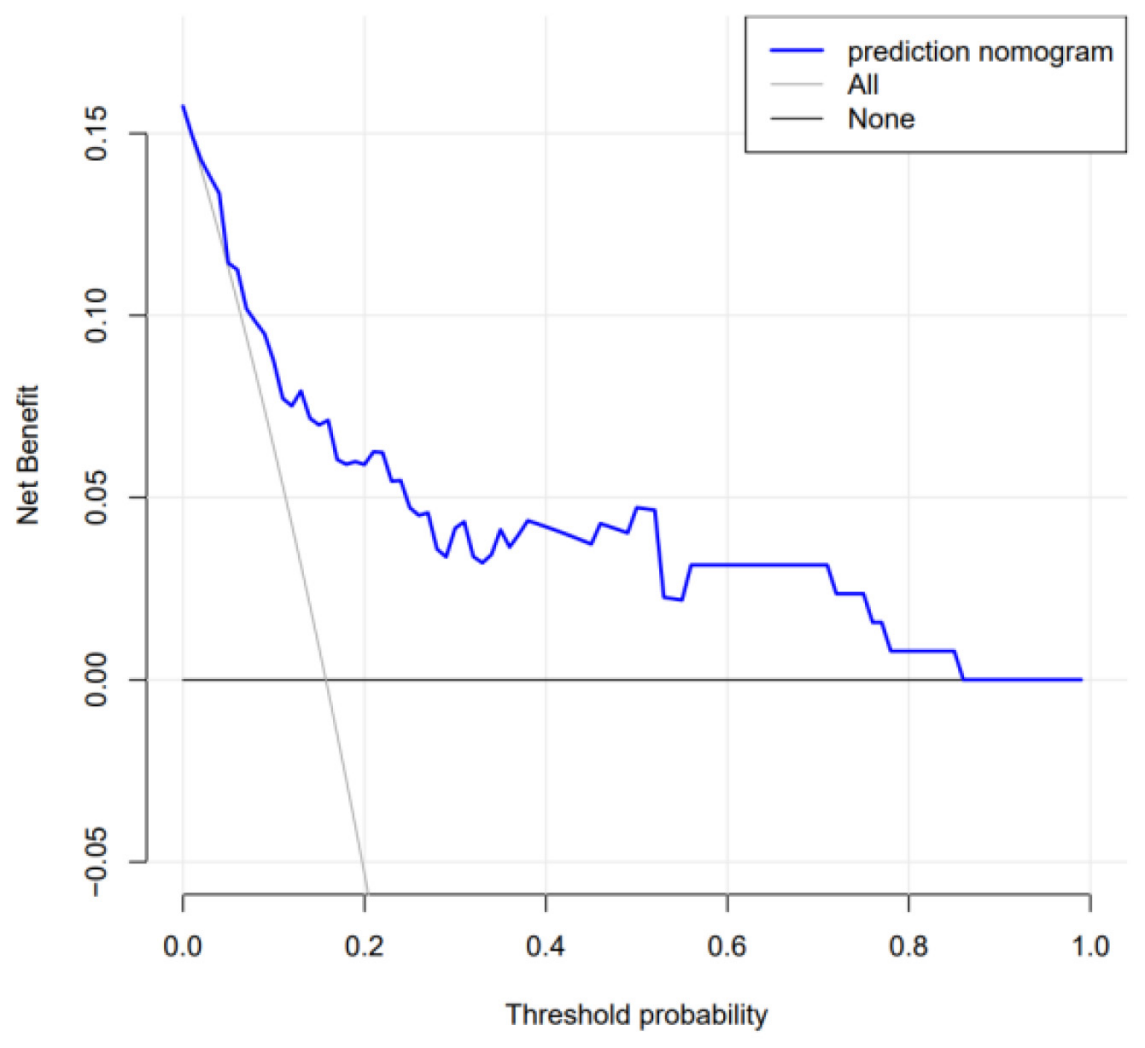
Decision curve analysis for the nomogram.

The DCA curve demonstrated a higher net benefit at a higher risk threshold probability compared to the baseline, confirming the clinical validity of the model (Figure 6).

## Discussion

Among the 127 cirrhotic patients with severe gastroesophageal varices or bleeding complications included in this study, 20 cases developed PVT during the 2-year follow-up, yielding an incidence of 15.75%. The average time to PVT occurrence after the first endoscopic variceal treatment was 12.45 ± 6.49 months. Of the PVT patients, 14 had partial PVT, and 6 had mural PVT, with all cases being asymptomatic. The portal vein and its branches were the most commonly affected sites, observed in 13 cases (65%). This study identified the inflammatory marker MLR, MELD score, baseline spleen diameter, and gastric varices sandwich therapy as independent factors influencing the occurrence of PVT. A prospective, single-center cohort study reported a PVT incidence of 6% in cirrhotic patients over 3 years.^21^ This cohort primarily included CTP grade A and B patients, with a majority having viral hepatitis or alcoholic liver disease, similar to the study population. In this study, 12.5% of patients underwent endoscopic ligation, while the remainder did not receive endoscopic or surgical intervention. The endoscopic treatments used included ligation and the sandwich method, with a PVT incidence of 15.75% within 2 years, higher than the 6% reported in the aforementioned study. Previous research has indicated that endoscopic ligation and sclerotherapy are independent risk factors for PVT in cirrhotic patients. ^3^^, ^^22^ One study found a 37.88% incidence of PVT within 1 year in cirrhotic patients with gastric variceal bleeding who underwent their first endoscopic sclerotherapy, with the dose of a sclerosing agent identified as an independent risk factor for PVT.^23^ These findings suggest that endoscopic variceal treatments may contribute to a higher incidence of PVT.

This study highlighted MLR as an influential baseline factor for PVT development, with an odds ratio (OR) of 23.540. Monocyte-to-lymphocyte ratio has increasingly been recognized as a risk factor for venous thrombosis. Prior studies have found that both MLR and PLR are associated with PVT in cirrhotic patients post-splenectomy and pericardial dissection.^15^ Monocyte-to-lymphocyte ratio, easily derived from routine blood tests, exhibited the highest specificity (92.5%) compared to MELD score and spleen diameter in predicting PVT, making it a valuable tool for early clinical screening and identifying high-risk PVT patients.

Spleen enlargement serves as a significant indicator of portal hypertension in cirrhosis, and several studies have shown that prediction models based on spleen diameter can forecast the occurrence of red signs in esophageal varices among cirrhotic patients.^24^ Prospective studies have identified several factors associated with severe portal hypertension, such as reduced white blood cell count, decreased portal blood flow velocity, and a history of gastroesophageal variceal bleeding, as risk factors for PVT in cirrhotic patients.^21^ In the present study, spleen diameter was identified as an independent factor influencing PVT occurrence after endoscopic variceal treatment, with an OR of 1.576. Consistent with these findings, domestic studies have shown that preoperative spleen diameter is a standalone risk factor for PVT following gastric variceal tissue glue injection. ^23^^, ^^25^ Additiona lly, larger spleen volume in patients with cirrhotic portal hypertension has been associated with a higher risk of PVT after splenectomy.^26^

The MELD score in the PVT group was lower than that in the non-PVT group, potentially linked to alcoholic cirrhosis. The MELD score, composed of TBIL, INR, and Cr, alongside the cause of cirrhosis, was analyzed. No significant differences were observed in TBIL, INR, or Cr levels between the groups, nor were there differences in CTP grading or CTP score, aligning with the findings of Xu Danqing. ^5^^, ^^15^^, ^^27^ The incid ence of alcoholic liver disease was notably higher in the PVT group compared to the non-PVT group, and this difference was statistically significant. Alcoholic liver disease has been shown to lower the MELD score, contributing to the lower MELD score observed in the PVT group. A cross-sectional study confirmed alcoholic liver disease as an influencing factor for PVT in cirrhotic patients,^5^ consistent with these findings. Alcohol consumption can inhibit fibrin dissolution,^28^ decreases nitric oxide (NO) synthesis, and cause vascular endothelial cell dysfunction, ^29^^, ^^30^ leading t o vasospasm or thrombosis.

In this study, the cut-off values and AUC were calculated for 3 continuous variables—MELD score, MLR, and spleen diameter—to predict PVT occurrence. The cut-off values were 9.5 for MELD score, 0.475 for MLR, and 17.135 cm for spleen diameter, with corresponding AUC values of 0.682, 0.647, and 0.688, respectively. The risk of PVT was higher when MELD score was below 9.5, MLR exceeded 0.475, and spleen diameter was larger than 17.135 cm. When combined, these 3 variables yielded the highest AUC value of 0.782, with the highest sensitivity (70%) and the highest specificity for MLR (92.5%).

This study identified gastric varices sandwich therapy as a risk factor for the occurrence of PVT, with an OR of 16.617. The sandwich therapy method involves the use of lauromacrogol and cyanoacrylate, with lauromacrogol acting as a sclerosing agent and cyanoacrylate as a tissue adhesive. Both substances cause endothelial damage, which contributes to PVT formation. A comparative study of 34 patients treated with tissue glue for gastric varices reported that one patient developed acute superior mesenteric vein thrombosis post-procedure.^31^ Additionally, a retrospective study found endoscopic sclerotherapy to be a significant risk factor for PVT in cirrhotic patients.^3^ Another study reported a 37.88% incidence of PVT within 1 year following endoscopic sclerotherapy,^23^ which is significantly higher than the 15.75% incidence of PVT observed in this study over a 2-year follow-up. These findings suggest that the sandwich method notably increases the risk of PVT in patients with severe gastric varices.

In this research, the predictive value of inflammatory markers for PVT formation was explored, considering various factors such as general data, laboratory indicators, imaging results, and endoscopic treatment methods. A prediction model based on a nomogram incorporating inflammatory markers was constructed, yielding an AUC of 0.807, indicating good discrimination. The results of the Hosmer-Lemeshow test and calibration curve demonstrated that the nomogram exhibited good calibration. Furthermore, the clinical utility of the model was validated by the DCA curve, which showed strong clinical effectiveness. The nomogram is quantitative, intuitive, and practical, offering ease of use for clinicians. To reduce PVT occurrence, limiting alcohol consumption and reconsidering the use of sandwich therapy in treating severe gastric varices may be beneficial. However, this study has certain limitations, such as its single-center retrospective design, which warrants further prospective studies with expanded research centers and larger sample sizes.

Inflammatory marker MLR, MELD score, spleen diameter at baseline, and gastric varices sandwich therapy were identified to be independent risk factors for PVT formation in cirrhotic patients with severe gastroesophageal varices within 2 years after their first endoscopic treatment.

## Figures and Tables

**Table 1.** Clinical Characteristics of the Study Population at Baseline

Variables	PVT Group (n = 20)	Non-PVT Group (n = 107)	*Z*/*t*/*χ*^2^ Value	*P*
Age (years)	55.25 ± 10.58	55.59 ± 11.00	−0.127	.899
Sex [n (%)] Male Female	15 (75) 5 (25)	65 (60.75) 42 (39.25)	1.468	.226
Alcohol [n (%)] Yes No	9 (45) 11 (55)	39 (36.45) 68 (63.55)	0.524	.469
Smoking [n (%)] Yes No	12 (60) 8 (40)	36 (33.64) 71 (66.36)	1.504	.220
Etiology [n (%)]				
HBV	6 (30)	55 (51.4)	2.125	.145
HCV	1 (5)	11 (10.28)	0.105	.745
Alcoholic	8 (40)	17 (15.89)	4.765	.029
Autoimmune	4 (20)	21 (19.63)	0.000	1.000
Unknown	2 (10)	9 (8.41)	0.000	1.000
CTP level [n (%)]			2.879	.237
A B C	7 (35) 13 (65) 0 (0)	48 (44.86) 51 (47.66) 8 (7.48)		
CTP score	7 (6.00~8.75)	7 (6.00~9.00)	−0.847	.397
MELD score	9.0 (2.5~9.0)	10.0 (7.0~12.0)	−2.166	.030
Diabetes mellitus [n (%)] Yes No	2 (10) 18 (90)	29 (27.1) 78 (72.9)	1.825	.177
Hypertension [n (%)] Yes No	5 (25) 15 (75)	17 (15.89) 90 (84.11)	0.977	.323
SBP [n (%)]	0 (0)	2 (1.87)	–	1.000
HE	1 (5.00)	3 (2.80)	0.000	1.000
Ascites [n (%)] Yes No	14 (70) 6 (30)	67 (62.62) 40 (37.38)	0.398	.528
Purpose of first endoscopy [n (%)] Preventing first bleeding Control bleeding	4 (20) 16 (80)	35 (32.71) 72 (67.29)	1.279	.258

CTP, Child-Turcotte-Pugh; HE, hepatic encephalopathy; MELD score, model for end-stage liver disease score; PVT, portal vein thrombosis; SBP, spontaneous bacterial peritonitis.

**Table 2.** Comparison of the Number of Endoscopic Treatment Methods Between 2 Groups

Endoscopic Therapy	PVT Group (n = 20)	Non-PVT Group (n = 107)	*χ*^2^ Value	*P*
EVL [n (%)]	6 (30.00)	21 (19.63)	0.552	.457
EVL+EVS [n (%)]	1 (5.00)	13 (12.15)	–	.696
EVL+GVS [n (%)]	11 (55.00)	71 (66.36)	0.950	.330
GVS [n (%)]	2 (10.00)	2 (1.87)	–	.117

EVL, esophageal variceal ligation; EVS, esophageal variceal sandwich; GVS, gastric variceal sandwich; PVT, portal vein thrombosis.

**Table 3.** Comparison of Laboratory and Imaging Results Between 2 Groups at Baseline

Variables	PVT Group (n = 20)	Non-PVT Group (n = 107)	*Z*/*t* Value	*P*
WBC (×10^9^/L)	4.15 (3.52~5.32)	3.94 (2.70~5.74)	−0.543	.587
MO (×10^9^/L)	0.26 (0.22~0.37)	0.24 (0.15~0.34)	−0.368	.171
LYM (×10^9^/L)	0.68 (0.61~1.11)	0.78 (0.56~1.19)	−0.288	.773
NE (×10^9^/L)	2.83 (1.99~3.56)	2.50 (1.67~4.14)	−0.592	.554
PLT (×10^9^/L)	84.00 (54.25~95.00)	73.00 (53.00~94.00)	−0.252	.801
MLR	0.36 (0.27~0.49)	0.31 (0.23~0.36)	−2.111	.035
PLR	94.65 (70.81~124.78)	88 (60.64~125.74)	−0.371	.711
NLR	3.80 (2.44~5.81)	3.00 (2.13~4.74)	−0.741	.459
PNI	38.40 ± 7.50	37.02 ± 5.70	0.940	.349
SII	262.45 (142.05~391.75)	224.70 (139.08~342.98)	−0.695	.487
MPR	0.13 (0.09~0.19)	0.13 (0.10~0.20)	−0.073	.942
RPR	0.21 (0.15~0.31)	0.20 (0.16~0.28)	−0.060	.952
HGB (g/L)	100.40 ± 33.40	98.35 ± 26.91	0.301	.764
ALB (g/L)	34.07 ± 6.94	32.52 ± 5.74	1.073	.285
GLB (g/L)	27.66 ± 7.62	29.15 ± 7.76	−0.794	.429
TBIL (μmol/L)	21.30 (13.6~27.83)	22.80 (13.40~41.00)	−0.665	.506
Cr (μmol/L)	61.50 (47.00~68.75)	58.80 (49.00~69.00)	−0.262	.793
PT (seconds)	15.99 ± 1.82	16.51 ± 2.56	−0.862	.390
INR	1.30 ± 0.19	1.36 ± 0.29	−0.950	.344
Spleen diameter (cm)	17.17 (14.70~17.60)	15.38 (14.20~16.50)	−2.671	.008
Spleen thickness (cm)	5.50 (5.25~6.10)	5.25 (4.70~6.00)	−1.365	.172
Portal vein diameter (cm)	1.40 (1.30~1.48)	1.40 (1.30~1.40)	0.000	1.000

ALB, albumin; Cr, creatinine; GLB, globulin; INR, international normalized ratio; LYM, lymphocytes; MLR, monocyte-to-lymphocyte ratio; MO, monocytes; NE, neutrophils; NLR, neutrophil-to-lymphocyte ratio; PLR, platelet-to-lymphocyte ratio; PLT, platelets; PNI, prognostic nutritional index; PT, prothrombin time; PVT, portal vein thrombosis; SII, systemic immune-inflammation index; TBIL, total bilirubin; WBC, white blood cells.

**Table 4.** Multivariate Logistic Regression Analysis

Variables	B	SE	Wald	*P*	OR	95% CI	
MELD score	−.205	.068	9.102	.003	.815	.713	.931
MLR	**1.000**	**0.491**	**4.154**	**.042**	**2.718**	**1.039**	**7.108**
Spleen diameter(cm)	.452	.135	11.288	.001	1.572	1.207	2.046
GVS alone	2.936	1.209	5.898	.015	18.832	1.762	201.282

GVS, gastric variceal sandwich; MELD, model for end-stage liver disease score; MLR, monocyte-to-lymphocyte ratio; OR, odds ratio.

**Table 5.** Receiver Operating Characteristics Curve of Predictive Variables for Patients with Portal Vein Thrombosis

Variables	Cut-Off Value	AUC (95% CI)	Sensitivity (%)	Specificity (%)	*P*
Spleen diameter(cm)	17.135	0.688 (0.555-0.820)	65.0	81.0	.008
MLR	0.475	0.647 (0.505-0.789)	35.0	92.5	.037
MELD score	9.500	0.682 (0.566-0.798)	51.4	85.0	.010
Combination of 3 indicators		0.782 (0.682-0.883)	70.0	77.6	.000

AUC, area under the curve; MELD, model for end-stage liver disease score; MLR, monocyte-to-lymphocyte ratio.

## Data Availability

The data that support the findings of this study are available on request from the corresponding author.
